# Concordance in measurable residual disease result after first and second induction cycle in acute myeloid leukemia: An outcome- and cost-analysis

**DOI:** 10.3389/fonc.2022.999822

**Published:** 2022-10-10

**Authors:** Jesse M. Tettero, Waleed K. W. Al-Badri, Lok Lam Ngai, Costa Bachas, Dimitri A. Breems, Catharina H. M. J. van Elssen, Thomas Fischer, Bjorn T. Gjertsen, Gwendolyn N. Y. van Gorkom, Patrycja Gradowska, Marjolein J. E. Greuter, Laimonas Griskevicius, Gunnar Juliusson, Johan Maertens, Markus G. Manz, Thomas Pabst, Jakob Passweg, Kimmo Porkka, Bob Löwenberg, Gert J. Ossenkoppele, Jeroen J. W. M. Janssen, Jacqueline Cloos

**Affiliations:** ^1^ Department of Hematology, Amsterdam Univerisity Medical Centers location Vrije Universiteit Amsterdam, Amsterdam, Netherlands; ^2^ Cancer Center Amsterdam, Imaging and Biomarkers, Amsterdam, Netherlands; ^3^ Department of Hematology, Ziekenhuis Netwerk Antwerpen, Antwerp, Belgium; ^4^ Department of Internal Medicine, Division of Hematology, GROW-School for Oncology and Developmental Biology, Maastricht University Medical Center, Maastricht, Netherlands; ^5^ Department of Hematology and Oncology, Otto von Guericke University Hospital Magdeburg, Magdeburg, Germany; ^6^ Department of Medicine, Hematology Section, Haukeland University Hospital, Bergen, Norway; ^7^ The Dutch-Belgian Hemato-Oncology Cooperative Group (HOVON) Data Center, Department of Hematology, Erasmus Medical Center (MC) Cancer Institute, Rotterdam, Netherlands; ^8^ Department of Epidemiology and Data Science, Amsterdam Univerisity Medical Centers, location Vrije Universiteit Amsterdam, Amsterdam, Netherlands; ^9^ Hematology, Oncology, Transfusion Medicine Center, Vilnius University Hospital Santaros Klinikos and Vilnius University, Vilnius, Lithuania; ^10^ Department of Hematology, Skanes University Hospital, Lund, Sweden; ^11^ Department of Hematology, University Hospital Gasthuisberg, Leuven, Belgium; ^12^ Department of Medical Oncology and Hematology, University Hospital, Zurich, Switzerland; ^13^ Swiss Group for Clinical Cancer Research (SAKK), Bern, Switzerland; ^14^ Department of Medical Oncology, Inselspital, University Hospital, Bern, Switzerland; ^15^ Department of Hematology, University Hospital, Basel, Switzerland; ^16^ Department of Hematology, Helsinki University Hospital Cancer Center, Helsinki, Finland; ^17^ Department of Hematology, Erasmus University Medical Center (MC) and Erasmus MC Cancer Institute, Rotterdam, Netherlands

**Keywords:** acute myeloid leukemia, measurable residual disease (MRD), multiparameter flow cytometry (MFC), prognostic value, earlier detection, guided therapy

## Abstract

Measurable residual disease (MRD) measured using multiparameter flow-cytometry (MFC) has proven to be an important prognostic biomarker in acute myeloid leukemia (AML). In addition, MRD is increasingly used to guide consolidation treatment towards a non-allogenic stem cell transplantation treatment for MRD-negative patients in the ELN-2017 intermediate risk group. Currently, measurement of MFC-MRD in bone marrow is used for clinical decision making after 2 cycles of induction chemotherapy. However, measurement after 1 cycle has also been shown to have prognostic value, so the optimal time point remains a question of debate. We assessed the independent prognostic value of MRD results at either time point and concordance between these for 273 AML patients treated within and according to the HOVON-SAKK 92, 102, 103 and 132 trials. Cumulative incidence of relapse, event free survival and overall survival were significantly better for MRD-negative (<0.1%) patients compared to MRD-positive patients after cycle 1 and cycle 2 (*p ≤* 0.002, for all comparisons). A total of 196 patients (71.8%) were MRD-negative after cycle 1, of which the vast majority remained negative after cycle 2 (180 patients; 91.8%). In contrast, of the 77 MRD-positive patients after cycle 1, only 41 patients (53.2%) remained positive. A cost reduction of –€571,751 per 100 patients could be achieved by initiating the donor search based on the MRD-result after cycle 1. This equals to a 50.7% cost reduction compared to the current care strategy in which the donor search is initiated for all patients. These results show that MRD after cycle 1 has prognostic value and is highly concordant with MRD status after cycle 2. When MRD-MFC is used to guide consolidation treatment (allo vs non-allo) in intermediate risk patients, allogeneic donor search may be postponed or omitted after cycle 1. Since the majority of MRD-negative patients remain negative after cycle 2, this could safely reduce the number of allogeneic donor searches and reduce costs.

## Introduction

Acute myeloid leukemia (AML) is characterized by an abnormal proliferation of myeloid progenitor cells. AML is usually treated by two cycles of intensive induction chemotherapy (“3+7”), followed by post-remission consolidation therapy after achieving complete remission (CR) (1, 2). This may either be an allogeneic stem cell transplantation (allo-SCT), one or more cycles of conventional chemotherapy, or an autologous stem cell transplantation (auto-SCT). Choosing the appropriate consolidation treatment is based on estimations of risks of treatment related mortality versus mortality due to relapse of the disease. Commonly, a genetics-based risk classification (mainly the ELN-2017) is used to facilitate this assessment at the time of diagnosis (3, 4). For ELN intermediate risk patients, measurable residual disease (MRD) during therapy is increasingly used as an additional marker to further stratify consolidation choices (5–7). MRD measured *via* multiparameter flow cytometry (MFC), or molecularly, by either quantitative PCR based techniques or next generation sequencing is used to determine leukemic burden after initial treatment (8, 9). MFC-MRD is most frequently used as it is applicable for almost all AML patients (>90%). In HOVON-SAKK trials, a positive MRD result after induction chemotherapy is defined as ≥0.1% of CD45-expressing cells with a leukemia associated immunophenotype (LAIP) for MFC-MRD or, for AML with mutated NPM1, >10^−4^ NPM1 copies using reverse transcriptase polymerase chain reaction. MRD positivity is associated with a significantly increased risk of relapse, shorter event-free survival (EFS) and inferior overall survival (OS) (10–16). The ELN MRD working party recommends MFC-MRD assessment after induction, which is often after two cycles of chemotherapy, and is closest to the consolidation time point, but there is still debate about the optimal time point (8, 17, 18). Several publications have shown that MRD also has prognostic value after one cycle of chemotherapy (19–23). Having a prognostic marker determined earlier during therapy can be helpful for earlier consolidation therapy decisions and clarity towards the patient. This applies in particular to patients of the intermediate risk category, as in this category consolidation therapy is increasingly being guided by MRD results. The earlier clarity *via* a MRD result can be used to be more restrictive in performing allogeneic donor searches and change the current practice to only initiating a search for MRD-positive patients, which can subsequently lead to a cost reduction. Here, we evaluate the concordance of MRD status measured by MFC in AML patients where MRD was assessed at both time point after first and second cycle of induction chemotherapy. In addition, we calculated potential cost reductions by depending the initiation of HLA-typing and donor search on the MRD result after cycle 1 and comparing it to the current practice of as early as possible after diagnosis.

## Materials and methods

### Patients and treatment

Patients included for analysis were treated according to the HOVON-SAKK AML92, AML102, AML103 and AML132 trials (6, 24–26), who achieved CR after cycle 1 and had a valid MRD result after 1^st^ and 2^nd^ chemotherapy cycle. These trials consist of newly diagnosed AML (APL excluded) patients between the age of 18 and 65, except for the AML103 study which consisted of patients older than 65 who were fit enough for high dose chemotherapy. All patients younger than 65 years were given two cycles of standard intensive “3 + 7” regimens as initial induction therapy consisting of idarubicin for 3 days and cytarabine for 7 days (overview per study can be found in **Supplementary Table S1**). Consolidation therapy was based on the risk classification applicable at the time. Only for ELN-2017 intermediate risk patients in the AML132 trial, this choice was guided by the MRD result after cycle 2 (6). All studies were reviewed and approved by the ethics committees of the participating institutions and were conducted in accordance with the Declaration of Helsinki. All patients provided their written informed consent to participate in the study.

### Multiparameter flow cytometry MRD assessment

Immunophenotyping was performed in the same way across all studies as previously described (27). Flow cytometry was performed on a FACS CANTO (BD Biosciences, San Jose, CA, USA) for all studies with either 6- or 8-color antibody panels, consisting of four or five different tubes (for details see **Supplementary Table S2**) (28). These panels have CD45, CD34, CD117, CD13 and HLA-DR as backbone markers. Leukemic population comprises of CD45 expressing cells (WBC) in combination with a primitive marker (CD34, CD117) and myeloid markers (CD13, CD33, or HLA-DR). Additional markers are used to define the leukemia associated phenotype (LAIP, e.g. CD2, CD7, CD36, CD22, CD19, CD15, CD11b, CD14, CD56). MRD was assessed after cycle 1 and cycle 2 in patients in morphologic CR/CRi. MRD percentage was defined as the percentage of LAIP-positive cells of the total WBC (CD45-expressing) population. Both MRD assessment and gating strategy were comparable for all included studies and following a strict protocol as previously published (29, 30).

### Cost-effectiveness analysis

**Figure 1:**
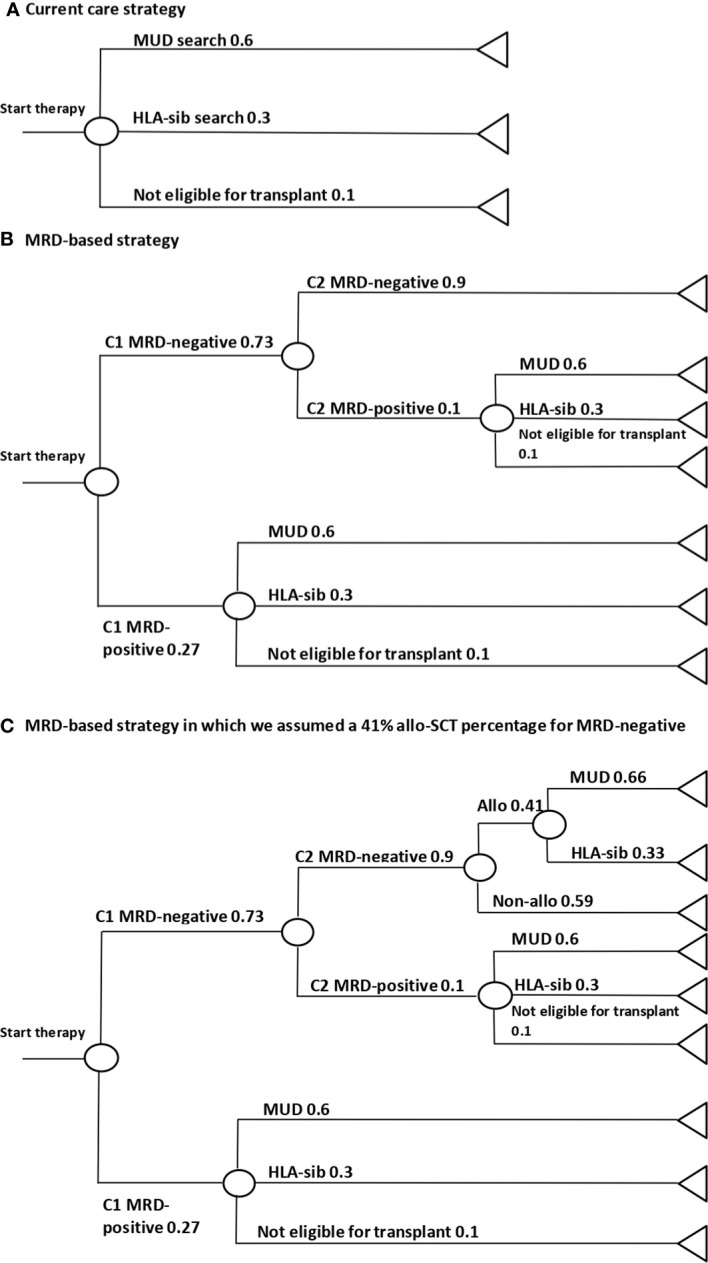
Decision trees of the three different strategies. **(A)** Current care strategy for intermediate risk patients were a donor is search is initiated for about 90% of patients of which 60% are match unrelated donors (MUD) and 30% siblings with HLA-match (HLA-sib). **(B)** MRD-based strategy were MRD-negative patients do not receive an allogeneic stem cell transplantation (allo-SCT). **(C)** Combination of MRD-based strategy with addition of treating physicians discretion, in which 41% of the MRD-negative patients after cycle 2 still receive an allo-SCT. This is the same allo-SCT percentage for MRD-negative patients as in this cohort.

We used decision trees to evaluate the impact of initiating the donor search based on the MRD result after cycle 1 on costs. We defined the following strategies: 1) the current care strategy with initiation of donor search for all patients at time of diagnosis; and 2) the MRD-based strategy with initiation of donor search based on MRD result after cycle 1 and no allo-SCT for MRD-negative patients. The decision trees are depicted in **Figure 1**. The probabilities of having a MRD-negative result after cycle 1 and cycle 2, and the availability of finding an HLA-matched donor or matched unrelated donor (MUD) were based on results from the included patients in this pooled set of patients and current practice (31, 32). Of the AML intermediate risk patients, we assumed to find a HLA-sibling match for approximately 30% of patients, MUD match for 60% of patients and no search for 10% because they are already deemed not fit for allo transplant. The HLA-sibling search was performed for more patients without a match, but these were not included in the cost analysis to keep it feasible. Furthermore, if a patient had a MRD-positive result after cycle 1, an search is initiated with the same ratio as the current strategy (60% MUD, 30% HLA-sib and 10% not eligible for transplant) and regardless of the status at a later time point.

We considered all costs related to the diagnostic process to find the right consolidation treatment, namely costs of the bone marrow (BM) aspiration and MRD measurement, HLA-typing and search for a suitable allo-SCT donor. An overview of the prices used can be found in **Supplementary Table S3**. Costs were based on the fixed tariffs negotiated between health insurers and hospitals from the Dutch Health Insurance Council and are from 2022 in euros (33, 34).

### Statistical analyses

Chi-square or Fisher exact test was used to assess differences at baseline for categorical variables, and the Mann-Whitney U test was used to analyze continuous variables. For cumulative incidence of relapse (CIR) a competitive risk framework was used with correction for competing risk (non-relapse mortality), where patients alive in continuing CR were censored at the date of last contact. EFS was defined as the time between MRD measurement after cycle 1 and the date of hematologic relapse or death. Overall survival was defined from the time of MRD measurement 1 until death from any cause or last follow-up. Survival differences were analyzed using the log-rank test and visualized with Kaplan-Meier curves for EFS and OS. Cox regression analysis was used to determine if MRD was independently associated with EFS and OS, both univariate- and multivariate. The proportional hazard assumption was tested on the basis of Schoenfeld residuals (35). Since the data consists of multiple clinical studies, we evaluated the heterogeneity between studies using the I^2^ statistic (36). All tests were two-tailed at a significance level of 0.05, unadjusted for multiplicity. Statistical analyses were performed using SPSS software (version 28; IBM Corporation, Armonk, NY) and the R software environment for statistical computing and graphics (version 4.0.3, Vienna, Austria) (37).

The expected costs of the two strategies were assessed using the decision trees of **Figure 1**. First, we calculated the average costs accumulated by a patient following a specific branch of the decision tree. Then, for each branch the unit costs were multiplied with the probability of a patient following a specific branch. Total cost per strategy were calculated by summing up the total expected costs of each branch and subsequently compared. To evaluate the impact of parameter uncertainty on the total expected costs, we conducted a probabilistic sensitivity analysis (PSA). A beta distribution was fitted to the MRD outcome parameter. For all other parameters, we assumed a 10% relative variance. Next, using Monte Carlo simulations, 1,000 draws were taken from these distributions. Uncertainty surrounding the expected costs was estimated using 95% credibility intervals (CrI) by estimating the 2.5% and 97.5% percentiles.

In addition, we conducted a threshold analysis to determine the maximum cost of the MRD measurement at which the total costs of the MRD-based strategy were equal to the current care strategy. Furthermore, we conducted a sensitivity analysis to assess if the MRD-based strategy would still be cost-efficient if physicians would deviate from the proposed non-allo consolidation treatment for MRD-negative patients. In this analysis, we assumed that the initiation of donor search was based on both MRD result after cycle 1 and treating physicians discretion. Based on the results of our cohort, we assumed that 41% of MRD-negative patients still received an allo-SCT despite ELN-2017 recommendation.

## Results

A total of 273 patients from the AML92 (34; 12.5%), AML102 (175; 64.1%), AML103 (12; 4.4%) and AML132 (52; 19%) trials met all inclusion criteria. The precise number of patients enrolled in different trials and reasons why patients were excluded in the present analysis can be found in **Supplementary Figure S1**. The analysis of heterogeneity for 5-year mortality demonstrated that trials are homogeneous (**Supplementary Figure S2**) with a percentage of heterogeneity on total variability (I^2^) of 0% (p=0.80). The baseline characteristics of the MRD-negative and MRD-positive patients after first and second induction cycle are shown in **Table 1**.

**Table 1:** Characteristics of patients by MRD-status after cycle 1 and cycle 2.

		MRD status after cycle 1			MRD status after cycle 2		
Characteristics		MRD-, N=196	MRD+, N=77	*p-value*	MRD-, N=216	MRD+, N=57	*p-value*
Age in 3 categories	<=45	47 (24%)	25 (32.5%)	0.108	54 (25%)	18 (31.6%)	0.558
	46-60	96 (49%)	27 (35.1%)		98 (45.4%)	25 (43.9%)	
	>60	53 (27%)	25 (32.5%)		64 (29.6%)	14 (24.6%)	
Sex	M	97 (49.5%)	38 (49.4%)	0.983	110 (50.9%)	25 (43.9%)	0.343
	F	99 (50.5%)	39 (50.6%)		106 (49.1%)	32 (56.1%)	
WHO performance status	WHO 0	101 (51.5%)	35 (45.4%)	0.066	107 (49.5%)	29 (50.9%)	0.044
	WHO 1	65 (33.2%)	24 (31.2%)		71 (32.9%)	18 (31.6%)	
	WHO 2	3 (1.5%)	6 (7.8%)		4 (1.9%)	5 (8.8%)	
WBC count at diagnosis	<20	104 (66.7%)	46 (70.8%)	0.566	116 (67.8%)	34 (68%)	0.993
	20-100	41 (26.3%)	13 (20%)		42 (24.6%)	12 (24%)	
	>100	11 (7.1%)	6 (9.2%)		13 (7.6%)	4 (8%)	
ELN-2017 risk	Favorable	87 (44.4%)	25 (32.5%)	0.092	92 (42.6%)	20 (35.1%)	0.165
	Intermediate	60 (30.6%)	22 (28.6%)		68 (31.5%)	14 (24.6%)	
	Adverse	48 (24.5%)	30 (39%)		55 (25.5%)	23 (40.4%)	
FLT3ITD x NPM1	Pos x pos	33 (16.8%)	7 (9.1%)	0.040	32 (14.8%)	8 (14%)	0.252
	Pos x Neg	17 (8.7%)	8 (10.4%)		20 (9.3%)	5 (8.8%)	
	Neg x pos	50 (25.5%)	10 (13%)		53 (24.5%)	7 (12.3%)	
	Neg x neg	81 (41.3%)	45 (58.4%)		96 (44.4%)	30 (52.6%)	
Consolidation treatment	None	20 (10.2%)	6 (7.8%)	0.172	23 (10.6%)	3 (5.3%)	0.070
	Cycle 3	63 (32.1%)	17 (22.1%)		65 (30.1%)	15 (26.3%)	
	Auto-HSCT	39 (19.9%)	14 (18.2%)		46 (21.3%)	7 (12.3%)	
	Allo-HSCT	74 (37.8%)	40 (51.9%)		82 (38%)	32 (56.1%)	

### MRD after cycle I

**Figure 2:**
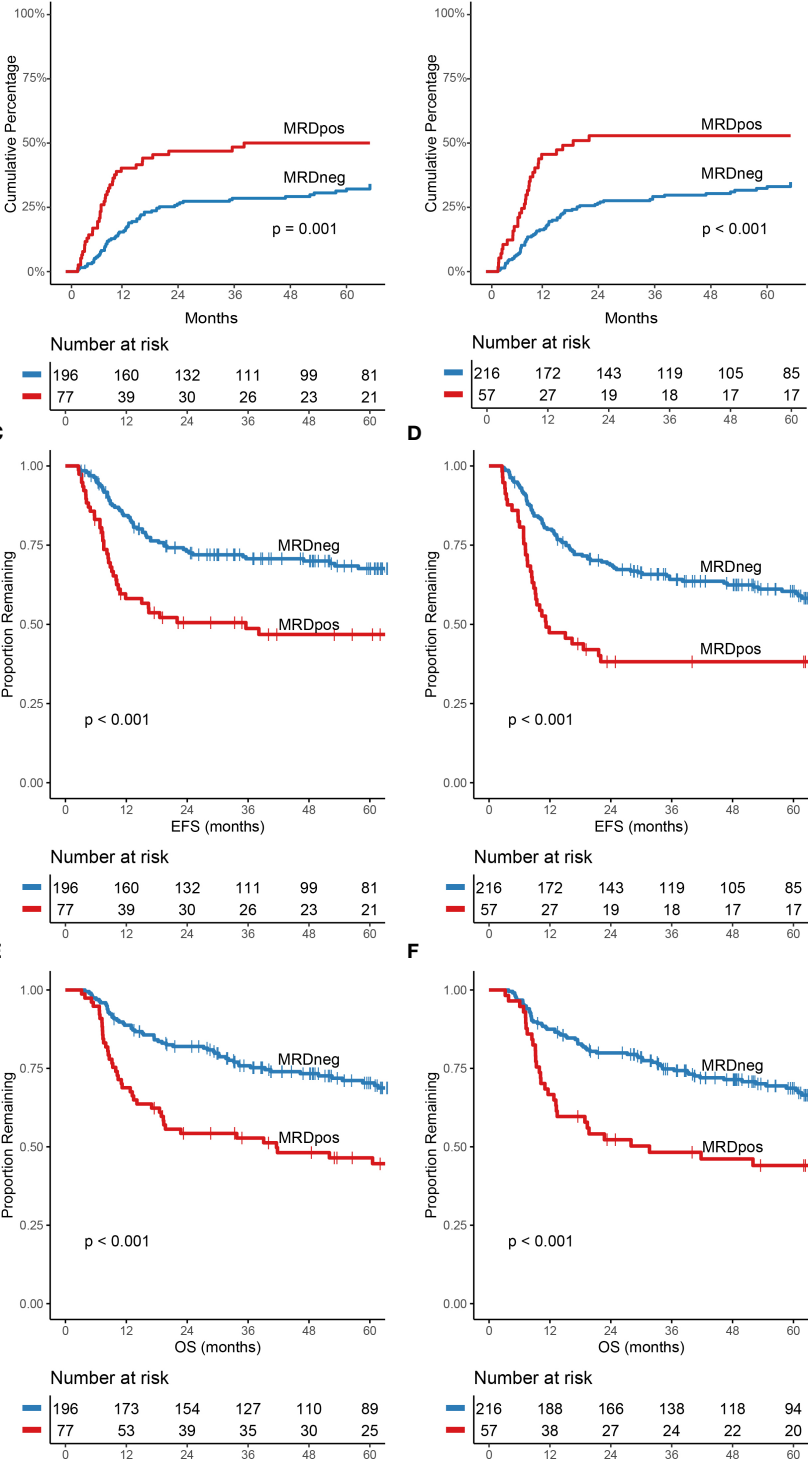
Cumulative incidence of relapse, event-free survival and overall survival stratified for MRD-status after cycle 1 and cycle 2. **(A)** CIR after cycle 1 and **(B)** CIR after cycle 2. **(C)** difference in EFS after cycle 1 and **(D)** after cycle 2. **(E)** OS difference for MRD status after cycle 1 and **(F)** after cycle 2. All curves were significantly different based on MRD-status (p<0.002).

Of the 273 patients who were in CR(i) and had a valid MRD result at both time points, 196 (72%) were MRD-negative after 1 cycle of chemotherapy and 77 (28%) patients were MRD-positive. A total of 38/77 (49.4%) of the MRD-positive patients relapsed at a median time of 8 months (range 2-38), compared to 62/196 (31.6%) of the MRD-negative patients with a median time of 13 months (range 2-82) (**Figure 2A**; Hazard Ratio (HR), 2.11; 95% CI, 1.41-3.16; P<0.001). At 5 years, MRD-positive patients both had a significantly worse EFS (**Figure 2C**; HR, 2.10; 95% CI, 1.46-3.02; P<0.001) and 5-year OS (45% for MRD-positive and 69% for MRD-negative patients (**Figure 2E**; HR, 2.12; 95% CI, 1.43-3.15; P<0.001)). MRD status after cycle 1 was significantly associated with FLT3-ITD/NPM1 status at diagnosis (**Table 1**). In univariate Cox regression analyses, age above 60 years at diagnose and ELN-2017 adverse risk was also significantly associated with worse EFS and OS (**Supplementary Table S4**). MRD-status after cycle 1 remained a significant prognostic factor in the multivariate model (p<0.001) along with age above 60 years at diagnosis and ELN-2017 adverse risk (**Supplementary Table S5**).

### MRD after cycle II

MRD-positive status after cycle 2 was significantly associated with the WHO performance status at diagnosis (**Table 1**). More patients were MRD-negative (216/273; 79.1%) compared to the time point after 1 cycle of chemotherapy. MRD-negative patients after cycle 2 had a significantly lower chance of relapsing in the first five years after therapy (**Figure 2B**; p<0.001) compared to MRD-positive patients. EFS (**Figure 2D**; HR, 2.03; 95% CI, 1.37-3.01; P=0.001) and OS (**Figure 2F**; HR, 2.02; 95% CI, 1.33-3.09; P=0.001) were also significantly better for patients who were MRD-negative after cycle 2. In multivariate Cox regression analyses, MRD-status remained a prognostic factor (p<0.001) for EFS and OS together with age above 60 years at diagnose and ELN-2017 adverse risk (**Supplementary Table S6**).

### Combining MRD after cycle 1 and cycle 2

**Figure 3:**
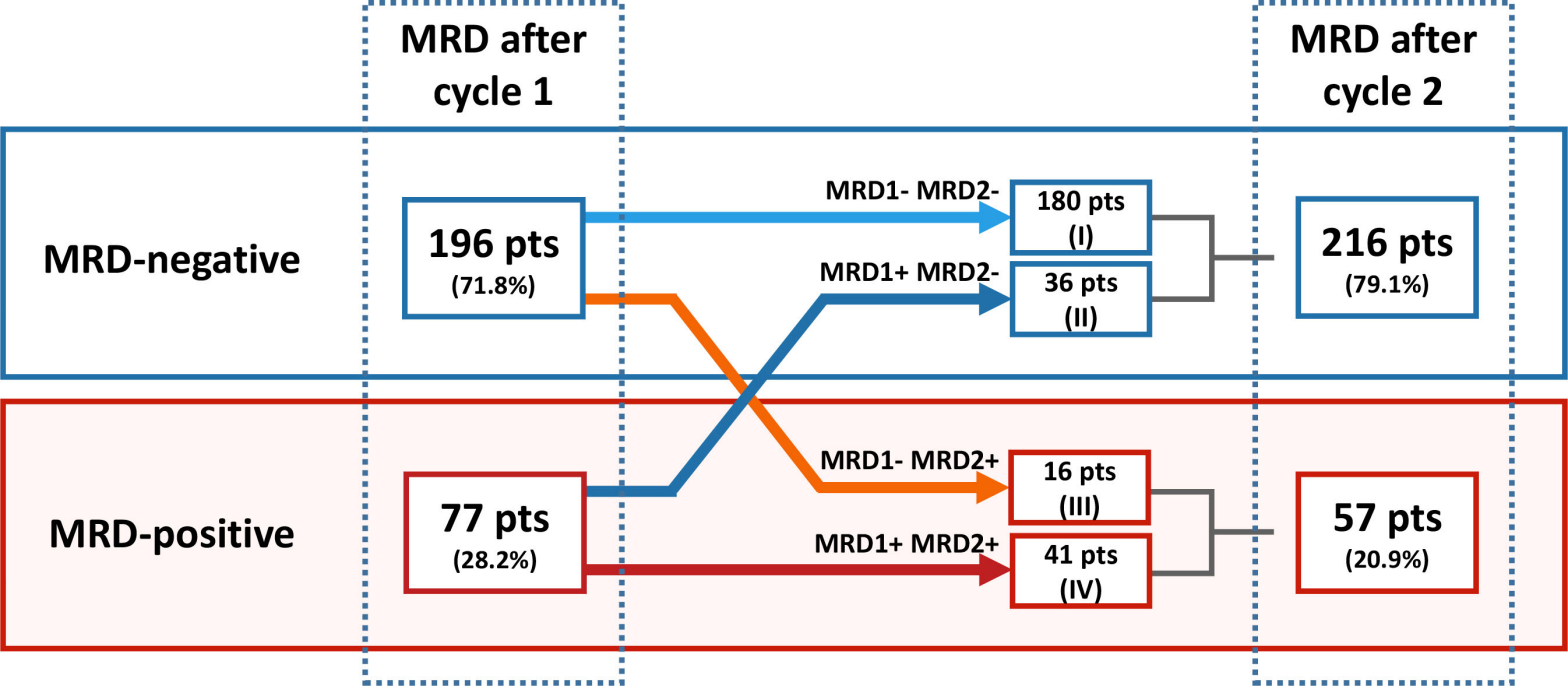
Fluctuations of MRD status between measurement after induction chemotherapy cycle I and the time point after chemotherapy cycle II. After one cycle of chemotherapy, 196 patients (71.8%) became MRD-negative and 77 patients were MRD-positive. After two cycles of chemotherapy, 216 patients (79.1%) were MRD-negative of which 180 were already MRD-negative after cycle 1 and 36 converted from MRD-positive to MRD-negative.

By combining the results of MRD after cycle 1 and cycle 2, we categorized the patients in four groups (**Figure 3**). 180 patients were MRD-negative at both time points (group I; MRD1-MRD2-), 36 patients were MRD-positive after cycle 1 and converted to MRD-negative (group II; MRD1+MRD2-), 16 patients were MRD-negative after cycle 1 and converted to MRD-positive after cycle 2 (group III; MRD1-MRD2+) and 41 patients were MRD-positive at both time points (group IV; MRD1+MRD2+). No distinct differences in baseline characteristics were found between the four groups (**Supplementary Table S7**). See **Figure 2** for an overview of the fluctuations of MRD status after combining the MRD results after cycle 1 and cycle 2. Of the 196 patients who were already MRD-negative after cycle 1, most remained negative after cycle 2 (180; 91.8%). This concordance was not found for MRD-positive patients, were 41 of the 77 MRD + patients after cycle 1 (53.2%) remained positive, whereas 36 patients converted to MRD-negativity. A higher MRD value after cycle 1 was associated with a higher chance of remaining MRD-positive at cycle 2, although no value could be found above which everyone remained MRD-positive. Of the 16 patients with an MRD value of 1.5% or higher after cycle 1, 11 (68.8%) remained positive after cycle 2 and this was 8/10 (80%) of the patients with an MRD level of 2.5% and higher.

**Figure 4:**
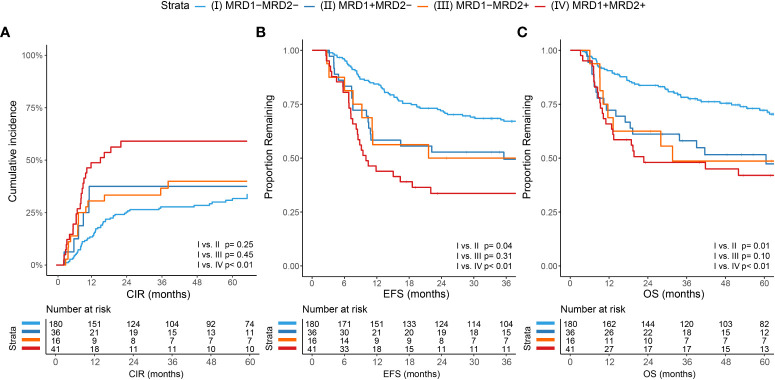
Cumulative incidence of relapse, event-free survival and overall survival compared for four groups based on combined MRD status of after cycle 1 and cycle 2. Four different groups were made based on the MRD status at time point after cycle 1 and cycle 2: (I) MRD negative at both time points (MRD1-MRD2-); (II) MRD-positive after cycle 1 and MRD-negative after cycle 2 (MRD1+MRD2-); (III) MRD-negative after cycle 1 and MRD-positive after cycle 2 (MRD1-MRD2+); and (IV) MRD-positive at both time points (MRD1+MRD2+). **(A)** Cumulative incidence of relapse (CIR) between the four groups with only a significant difference between MRD1-MRD2-(I) and MRD1+MRD2+(IV) (p<0.001). **(B)** Event-free survival (EFS) difference of the four groups with a significant difference between group (I) and group (IV) (p<0.001), but also between group (I) and group (II) (p=0.044). **(C)** Overall survival (OS) was also significantly different both between group (I) and group (IV) (p=0.001), and between group (I) and group (II) (p=0.014).

The cumulative incidence of relapse (CIR) was significantly different between MRD-negative patients at both time points (group I; MRD1-MRD2-) and patients who were MRD-positive at both time points (MRD1+MRD2+; p<0.001, **Figure 4A**). There was no significant difference between group I and patients who were positive at one of the two time points (MRD1+MRD2- and MRD1-MRD2+). For EFS, there was a difference between MRD1-MRD2- patients and MRD1+MRD2+ patients (p<0.001), but also between MRD1-MRD2- patients and MRD1+MRD2- (p=0.044, **Figure 4B**). These differences were also seen for OS with 73.9% of MRD1-MRD2- patients surviving five years after start of treatment compared to 52.8% of MRD1+MRD2- patients (p=0.014), 50% of MRD1-MRD2+ patients (not significant; p=0.100) and 43.9% of MRD1+MRD2+ patients (p=0.001, **Figure 4C**).

### Decision tree analysis

Of the 273 patients included, 82 were classified as ELN-2017 intermediate risk, of which 60 patients (73%) were MRD-negative after cycle 1 and 54 (54/60; 90%) of these remained negative after cycle 2. The decision trees of the two strategies and the sensitivity analysis are depicted in **Figure 1**. Using decision tree analyses, we calculated an expected total cost of €1,127,342 per 100 patients for the current care strategy, in which for 90% of patients an allogeneic donor search is initiated (**Figure 1A**). In the MRD-based strategy in which MRD-negative intermediate risk patients do not receive an allo-SCT, the search is not initiated for 65.7% of total intermediate risk patients with an expected cost of €555,591 per 100 patients (**Figure 1B**). This strategy results in a cost reduction of –€571,751 (95% CrI: –€705,309 to –€464,698) per 100 patients, which equals to a 50.7% reduction compared to the current care strategy. The PSA showed that the proposed MRD strategy was consistently cheaper compared to the current care strategy. The threshold analysis showed that the combined cost of the BM aspiration and MRD-measurement could increase to €7,406 (+438%), in order for the MRD-based strategy to be equally expensive as the current care strategy. The sensitivity analysis in which the choice to start a donor search is based on the MRD-result after cycle 1 and treating physicians discretion (third decision tree), resulted in 34.2% less initiation of donor searches with in an expected cost of €789,406 per 100 patients. This means a cost reduction of –€337,936 (95% CrI: –€470,207 to –€222,322) compared to the current care strategy (**Figure 1C**).

## Discussion

MRD-negative status after both one- and two cycles of chemotherapy was significantly associated with less chance of relapse, better EFS and OS (**Figure 2**; *p ≤* 0.002, for all comparisons). Curves on both time points had similar fits, which suggests similar prognostic value. Comparable results were found after grouping the patients based on the MRD results at both time points, where patients negative at both time points had a significantly better outcome (CIR, EFS and OS) compared to patients positive at both time points (**Figure 4**). Also evident was the difference in EFS (p=0.044) and OS (p=0.014) between patients who achieved MRD-negativity only after cycle 2 (MRD1+MRD2-) compared to patients who were MRD-negative after both cycles (MRD1-MRD2-). MRD-negative after cycle 1 and positive after cycle 2 (MRD1-MRD2+) was the least observed, with only 5.9% of patients. Likely due to the small sample size, this group was not significantly different from MRD1-MRD2- despite showing similar curves when compared to the MRD1+MRD2- subgroup. These results underline that MRD status after 1 cycle of chemotherapy has strong prognostic implication with failure to achieve MRD-negativity after 1 cycle being associated with a clearly worse outcome.

In addition, because a MRD-negative result after cycle 1 is highly concordant with a negative MRD result after cycle 2 of chemotherapy, it can be used to postpone the initiation of a transplant donor search for intermediate risk patients. This alternative strategy will result in a decrease in donor searches of between 34.2%-65.7% for intermediate risk patients and average cost savings of €571,751 per 100 patients. Therefore, the proposed alternative strategy can be considered as a valuable alternative approach, especially for countries with more limited budgets. However, a downside to a later search initiation is the potential delay of an allo-SCT in the 10% of MRD-negative patients after cycle 1 who do convert to MRD-positive after cycle 2. The sensitivity analysis showed that even with 41% of MRD-negative patients still receiving an initial allo-SCT, our proposed strategy would be more cost efficient. This analysis however, does not take into account the possible allo-SCT as second consolidation therapy needed after relapse. The decision tree strategy considers all other vital variables in our situation, but caution is warranted when results are being extrapolated to other countries as they could face different conditions.

Up to now, although the prognostic value of MRD after one cycle of chemotherapy has been demonstrated before, information about MRD concordance between the two time points has been sparse (19, 20, 23). One notable exception is the UK-NCRI AML17 study, which showed corresponding results in MRD concordance despite having slightly different inclusion criteria (NPM1+ patients were excluded) (20). The AML17 trial also showed a high degree of concordance between MRD-negative results at the two time points, with 90% of the patients achieving MRD-negativity after cycle 1 remaining MRD-negative after cycle 2. Furthermore, this study also showed the lack of concordance between MRD-positive results at the two time points, with almost 50% conversion from MRD-positive after cycle 1 to MRD-negative after cycle 2, which even more suggests that the second cycle of chemotherapy is an important part of the treatment sequence in these patients.

In general, MRD is not routinely measured after one cycle of chemotherapy since centers have less experience with this time point and it is not generally recommended by the ELN MRD working party (17). Our study only included patients who had a valid MRD measurement after 1 and 2 cycles of chemotherapy, which means that all patients had to be in CR after cycle 1. As a result, conclusions from this study cannot be translated to all AML patients but only to patients already in CR after cycle 1. Moreover, since MRD was not systematically collected after 1 cycle of chemotherapy, relatively many patients were not eligible for inclusion in our study and this could potentially form a selection bias.

Measuring MRD after one cycle of induction chemotherapy has the benefit of giving prognostic value at an early stage of therapy and due to the high concordance with the measurement after cycle 2, a high degree of clarity for the recommended consolidation therapy in the case of an intermediate risk patient. Therefore, we would recommend to incorporate this time point into upcoming studies. However, given the limited experience with measuring MRD after cycle 1, we do not value this point as a replacement for the current “gold standard” after two cycles of chemotherapy. The high degree of concordance between MRD-negativity between the two time points signifies the question if adverse risk patients who reach MRD-negativity after cycle 1, do still benefit from the second induction course or whether they should immediately proceed to transplantation if a donor is available (38). Future (randomized) studies to address this hypothesis are warranted. In addition, when opting for allo-SCT, the risk for nonrelapse mortality is an important factor that needs to be considered next to the ELN risk classification and MRD status (39).

In conclusion, our findings highlight two facets of measuring MFC-MRD after one cycle of chemotherapy. First, achieving MRD-negative CR after one cycle of chemotherapy gives a prognostic advantage in terms of EFS and OS compared to patients who are in CR but are MRD-positive or who are persistent MRD-positive at both time points. Secondly, there is a high concordance between MRD-negative result after cycle 1 and cycle 2 which can be used to pre-sort intermediate risk patient sooner to a recommended consolidation therapy. The early time point of response data can be used to postpone or omit the search for an allogeneic donor, which will result in a cost-reduction and provide patients with more certainty about the course of their further treatment.

## Data availability statement

The raw data supporting the conclusions of this article contain too much identifiable data that it will not be made available by the authors. Requests to access the datasets should be directed to j.cloos@amsterdamumc.nl.

## Ethics statement

The studies involving human participants were reviewed and approved by Medical Ethical Committee Erasmus MC. The patients/participants provided their written informed consent to participate in this study.

## Author contributions

JT, WA-B, CE, GG, JJ, and JC contributed to conception and design of the study. DB, TF, BG, LG, GJ, JM, MM, TP, JP, KP, BL, GO, and JJ collected the data. JT and WA-B organized the database. JT and MG performed the statistical analysis. PG provided statistical consultation. JT wrote the first draft of the manuscript. All authors contributed to manuscript revision, read, and approved the submitted version.

## Conflict of interest

The authors declare that the research was conducted in the absence of any commercial or financial relationships that could be construed as a potential conflict of interest.

## Publisher’s note

All claims expressed in this article are solely those of the authors and do not necessarily represent those of their affiliated organizations, or those of the publisher, the editors and the reviewers. Any product that may be evaluated in this article, or claim that may be made by its manufacturer, is not guaranteed or endorsed by the publisher.
