# Supplementary material for: Concordance in measurable residual disease result after first and second induction cycle in acute myeloid leukemia: An outcome- and cost-analysis
Source: Front Oncol. 2022 Oct 10;12:999822. doi: 10.3389/fonc.2022.999822 (PMC9589259; doi:10.3389/fonc.2022.999822)
Supplement: Supplementary file 1 [file DataSheet_1.pdf]

Tettero et al. Supplementary Material

Table S1: Overview of induction cycle I and cycle II per included trial

Study	Induction cycle I	Induction cycle II
**AML92**	**Idarubicin** 12mg/m2 for 3 days and **cytarabine** 200mg/m2 for 7 days (with or without laromustine on day 2)	**Amsacrine** 120mg/m2 for 3 days and **cytarabine** 1g/m2 twice daily on days 1-6 (with or without laromustine on day 2)
**AML102**	**Idarubicin** 12 mg/m2 for 3 days and **cytarabine** 200 mg/m2 for 7 days (with or without the addition of clofarabine at 10 mg/m2 for 5 days)	**Amsacrine** 120 mg/m2 for 3 days and **cytarabine** 1g/m2 twice daily on days 1-6 (with or without the addition of clofarabine 10 mg/m2 for 5 days)
**AML103**	**Daunorubicin** 60 mg/m2 for 3 days and **cytarabine** 1g/m2 twice daily on days 1-6 (with or without the addition of lenalidomide/Tosedostat on days 1-21)	**Cytarabine** 1g/m2 twice daily on days 1-6 (with or without the addition of lenalidomide/Tosedostat on days 1-21
**AML132**	**Idarubicin** 12 mg/m2 for 3 days and **cytarabine** 200 mg/m2 for 7 days (with or without lenalidomide 10/15mg on days 1-21)	**Daunorubicin** 60 mg/m2 for 3 days and **cytarabine** 1g/m2 twice daily on days 1-6 (with or without the addition of lenalidomide 10/15mg on days 1-21)

Table S2A: 6-color antibody panel

Tube	FITC	PE	PerCP	PC7	APC	APC-H7
**1**	PBS	PBS	CD45	CD34	PBS	PBS
**2**	CD2	CD7	CD45	CD34	CD13	HLA-DR
**3**	CD36	CD133	CD45	CD34	CD22	CD19
**4**	CD15	CD33	CD45	CD34	CD11b	CD14
**5**	CD13	CD56	CD45	CD34	CD117	HLA-DR

Table S2B: 8-color antibody panel in 5 tubes

Tube	FITC	PE	PerCP-CY5.5	PC7	APC	APC-H7	HV450	KO
**1**	HLA-DR	CD33	CD13	PBS	CD14	PBS	CD34	CD45
**2**	CD44	CLL-1/ CLEC12a	CD13	CD56	CD38	HLA-DR	CD34	CD45
**3**	CD7	TIM-3	CD13	CD117	CD38	CD19	CD34	CD45
**4**	CD2	CD133	CD13	CD117	CD38	CD19	CD34	CD45
**5**	CD36	CD123	CD13	CD33	CD38	CD14	CD34	CD45

Table S2C: 8-color antibody panel in 4 tubes

Tube	FITC	PE	PerCP-CY5.5	PC7	APC	APC-H7	BV421	HV500c
**1**	CD7	CD56	CD34	CD117	CD33	HLA-DR	CD13	CD45
**2**	CD15	CD22	CD34	CD117	CD19	HLA-DR	CD13	CD45
**3**	CD36	CD14	CD34	CD117	CD11b	HLA-DR	CD13	CD45
**4**	CD2	CD133	CD34	CD117	CD33	HLA-DR	CD13	CD45

Table S3: Cost prices used. All prices are fixed tariffs in euros negotiated between health insurers and hospitals from the Dutch Health Insurance Council from 2022 in Euros

Variable	Medical description of product	Cost
**BM+MRD**	Bone marrow aspiration done by a hematologist with total cost of MRD measurement via flow cytometry	€ 1,689.13
**MUD**	Search without purchase of match unrelated donor (MUD) used for allogeneic stem cell transplantation (allo-SCT)	€ 16,371.99
**HLA-sib**	Search without harvesting of stem cells of HLA-identical sibling (HLA-sib) donor	€ 4,834.08

Table S4A: Univariate testing of predictors for EFS

Event Free Survival						
**Variables**	**N**		**Coefficient**	**SE**	**95% CI**	**P Value**
**Age in 3 categories**	72	<=45				
	123	46-60	1.240	0.238	0.778-1.977	0.366
	78	>60	1.679	0.250	1.029-2.740	0.038
**Sex**	135	Male				
	138	Female	0.740	0.181	0.519-1.054	0.095
**WHO performance status**	136	WHO 0				
	89	WHO 1	1.257	0.202	0.847-1.866	0.256
	9	WHO 2	1.221	0.518	0.443-3.370	0.700
**WBC count at diagnosis**	150	<20				
	54	20-100	1.278	0.226	0.820-1.990	0.278
	17	>100	1.259	0.375	0.604-2.624	0.539
**ELN-2017 risk score**	112	Favorable				
	82	Intermediate	1.121	0.231	0.714-1.762	0.620
	78	Adverse	1.958	0.210	1.297-2.957	0.001
**FLT3ITD x NPM1**	40	Pos x pos				
	25	Pos x neg	1.631	0.372	0.787-3.380	0.189
	60	Neg x pos	1.006	0.324	0.532-1.899	0.987
	126	Neg x neg	1.267	0.288	0.720-2.229	0.412
**Consolidation treatment**	26	None				
	80	Cycle 3	1.286	0.369	0.624-2.651	0.495
	53	Auto-SCT	1.024	0.396	0.471-2.224	0.953
	114	Allo-SCT	1.261	0.361	0.621-2.558	0.521
**Flow MRD status after cycle 1**	196	Neg				
	77	Pos	2.097	0.186	1.456-3.018	0.000
**Flow MRD status after cycle 2**	216	Neg				
	57	Pos	2.031	0.201	1.371-3.010	0.000

Auto-SCT, Autologous stem cell transplantation; Allo-SCT, Allogeneic stem cell transplant; ELN, European Leukemia-NET; Neg, negative; Pos, positive; SE, standard error; CI, confidence interval; WBC, white blood cell count; WHO, world health organization.

Table S4B: Univariate testing of predictors for OS

Overall Survival						
**Variables**	**N**		**Coefficient**	**SE**	**95% CI**	**P Value**
**Age in 3 categories**	72	<=45				
	123	46-60	1.376	0.275	0.803-1.376	0.246
	78	>60	2.308	0.280	1.332-3.999	0.003
**Sex**	135	Male				
	138	Female	0.721	0.199	0.488-1.064	0.098
**WHO performance status**	136	WHO 0				
	89	WHO 1	1.293	0.224	0.834-2.005	0.251
	9	WHO 2	1.606	0.522	0.577-4.473	0.364
**WBC count at diagnosis**	150	<20				
	54	20-100	1.324	0.247	0.816-2.148	0.255
	17	>100	1.082	0.432	0.464-2.521	0.855
**ELN-2017 risk score**	112	Favorable				
	82	Intermediate	1.206	0.268	0.714-2.037	0.484
	78	Adverse	2.842	0.231	1.806-4.473	0.000
**FLT3ITD x NPM1**	40	Pos x pos				
	25	Pos x neg	1.634	0.409	0.733-3.642	0.230
	60	Neg x pos	1.012	0.359	0.500-2.047	0.973
	126	Neg x neg	1.268	0.320	0.677-2.376	0.458
**Consolidation treatment**	26	None				
	80	Cycle 3	0.768	0.365	0.375-1.571	0.469
	53	Auto-SCT	0.668	0.404	0.303-1.472	0.317
	114	Allo-SCT	0.935	0.349	0.472-1.852	0.846
**Flow MRD status after cycle 1**	196	Neg				
	77	Pos	2.124	0.202	1.430-3.153	0.000
**Flow MRD status after cycle 2**	216	Neg				
	57	Pos	2.023	0.216	1.326-3.086	0.001

Auto-SCT, Autologous stem cell transplantation; Allo-SCT, Allogeneic stem cell transplant; ELN, European Leukemia-NET; Neg, negative; Pos, positive; SE, standard error; CI, confidence interval; WBC, white blood cell count; WHO, world health organization.

Table S5A: Multivariate model event free survival / MRD after cycle 1

EFS - MRD 0.1% cut-off after cycle 1						
**Variables**	**N**		**Coefficient**	**SE**	**95% CI**	**P Value**
**MRD status after cycle 1**	77	Pos	2.051	0.189	1.417-2.968	0.000
**Age in 3 categories**	72	<=45				
	123	46-60	1.536	0.243	0.950-2.462	0.081
	78	>60	1.723	0.250	1.055-2.815	0.030
**ELN-2017 risk score**	112	Favorable				
	82	Intermediate	1.110	0.232	0.705-1.749	0.651
	78	Adverse	1.901	0.216	1.246-2.901	0.003

Table S5B: Multivariate model overall survival / MRD after cycle 1

OS - MRD 0.1% cut-off after cycle 1						
**Variables**	**N**		**Coefficient**	**SE**	**95% CI**	**P Value**
**MRD status after cycle 1**	77	Pos	2.109	0.204	1.413-3.148	0.000
**Age in 3 categories**	72	<=45				
	123	46-60	1.800	0.279	1.041-3.111	0.035
	78	>60	2.389	0.281	1.377-4.148	0.002
**ELN-2017 risk score**	112	Favorable				
	82	Intermediate	1.234	0.268	0.730-2.087	0.432
	78	Adverse	2.772	0.236	1.745-4.405	0.003

Table S6A: Multivariate model event free survival / MRD after cycle 2

EFS - MRD 0.1% cut-off after cycle 2						
**Variables**	**N**		**Coefficient**	**SE**	**95% CI**	**P Value**
**MRD status after cycle 2**	57		2.068	0.202	1.391-3.073	0.000
**Age in 3 categories**	72	<=45				
	123	46-60	1.453	0.241	0.905-2.332	0.122
	78	>60	1.790	0.251	1.094-2.929	0.020
**ELN-2017 risk score**	112	Favorable				
	82	Intermediate	1.111	0.232	0.705-1.750	0.650
	78	Adverse	1.924	0.216	1.260-2.937	0.002

Table S6B: Multivariate model overall survival / MRD after cycle 2

**OS - MRD 0.1% cut-off after cycle 2**						
**Variables**	**N**		**Coefficient**	**SE**	**95% CI**	**P Value**
**MRD status after cycle 1**	77	Pos	2.171	0.218	1.416-3.329	0.000
**Age in 3 categories**	72	<=45				
	123	46-60	1.709	0.278	0.992-2.945	0.053
	78	>60	2.469	0.283	1.419-4.297	0.001
**ELN-2017 risk score**	112	Favorable				
	82	Intermediate	1.216	0.268	0.719-2.057	0.465
	78	Adverse	2.785	0.237	1.751-4.430	0.000

Table S7: Baseline characteristics of four groups categorized by the combined MRD results from after cycle 1 and after cycle 2

Variables		I (MRD1- MRD2-)		II (MRD1+ MRD2-)		III (MRD1- MRD2+)		IV (MRD1+ MRD2+)		Total	
		Count	Column N %	Count	Column N %	Count	Column N %	Count	Column N %	Count	Column N %
**Age**	<= 45	44	24.4%	10	27.8%	3	18.8%	15	36.6%	72	26.4%
	46-60	88	48.9%	10	27.8%	8	50.0%	17	41.5%	123	45.1%
	>60	48	26.7%	16	44.4%	5	31.3%	9	22.0%	78	28.6%
**Sex**	Male	90	50.0%	20	55.6%	7	43.8%	18	43.9%	135	49.5%
	Female	90	50.0%	16	44.4%	9	56.3%	23	56.1%	138	50.5%
**WHO performance status**	WHO 0	89	49.4%	18	50.0%	12	75.0%	17	41.5%	136	49.8%
	WHO 1	62	34.4%	9	25.0%	3	18.8%	15	36.6%	89	32.6%
	WHO 2	3	1.7%	1	2.8%	0	0%	5	12.2%	9	3.3%
	Unknown	26	14.4%	8	22.2%	1	6.3%	4	9.8%	39	14.3%
**WBC-count x 10^9 L**	<=20	95	66.4%	21	75.0%	9	69.2%	25	67.6%	150	54.9%
	20-100	37	25.9%	5	17.9%	4	30.8%	8	21.6%	54	19.8%
	>100	11	7.7%	2	7.1%	0	0.0%	4	10.8%	17	6.2%
**ELN-2017 risk score**	Favorable	82	45.6%	10	27.8%	5	31.3%	15	36.6%	112	41.0%
	Intermediate	54	30.0%	14	38.9%	6	37.5%	8	19.5%	82	30.0%
	Adverse	43	23.9%	12	33.3%	5	31.3%	18	43.9%	78	28.6%
**FLT3-ITD x NPM1**	neg x neg	75	41.7%	21	58.3%	6	37.5%	24	58.5%	126	46.2%
	pos x neg	14	7.8%	6	16.7%	3	18.8%	2	4.9%	25	9.2%
	neg x pos	48	26.7%	5	13.9%	2	12.5%	5	12.2%	60	22.0%
	pos x pos	29	16.1%	3	8.3%	4	25.0%	4	9.8%	40	14.7%
	n.a.	14	7.8%	1	2.8%	1	6.3%	6	14.6%	22	8.1%
**Consolidation treatment**	None	19	10.6%	4	11.1%	1	6.3%	2	4.9%	26	9.5%
	Cycle 3	59	32.8%	6	16.7%	4	25.0%	11	26.8%	80	29.3%
	Auto-SCT	37	20.6%	9	25.0%	2	12.5%	5	12.2%	53	19.4%
	Allo-SCT	65	36.1%	17	47.2%	9	56.3%	23	56.1%	114	41.8%

Auto-SCT, Autologous stem cell transplantation; Allo-SCT, Allogeneic stem cell transplant; ELN, European Leukemia-NET; Neg, negative; Pos, positive; WBC, white blood cell count; WHO, world health organization.

Supplementary Figures

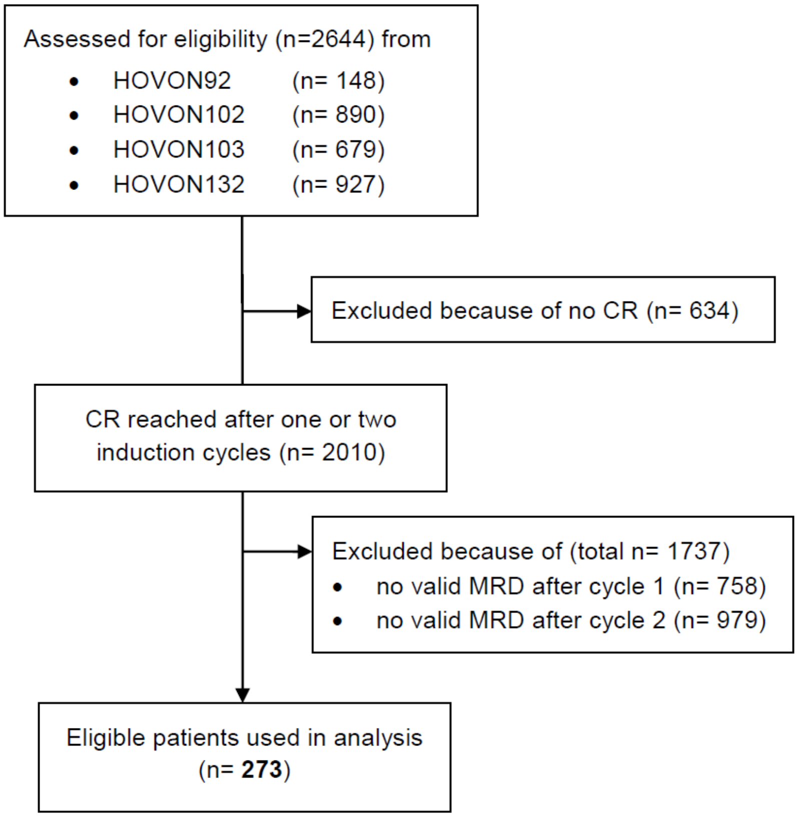

Figure S1: Patient flow chart. Patients with AML, included in four consecutive HOVON-SAKK trials, who were eligible for the present analysis with a valid MRD result after first- and second induction chemotherapy.

	MRD-neg		MRD-pos			Risk ratio
**Study or Subgroup**	**Events**	**Total**	**Events**	**Total**	**Weight**	**M-H, Random, 95% CI**
AML92	7	20	10	14	20.9%	0.49 [0.25 , 0.97]
AML102	50	141	17	34	59.9%	0.71 [0.47 , 1.06]
AML103	6	11	1	1	10.7%	0.72 [0.28 , 1.88]
AML132	9	44	3	8	8.5%	0.55 [0.19 , 1.59]
**Total (95% CI)**		**216**		**57**	**100.0%**	**0.64 [0.47 , 0.88]**
Total events:	72		31			

Heterogeneity: Tau² = 0.00; Chi² = 0.99, df = 3 (P = 0.80); I² = 0%
Test for overall effect: Z = 2.77 (P = 0.006)
Test for subgroup differences: Not applicable

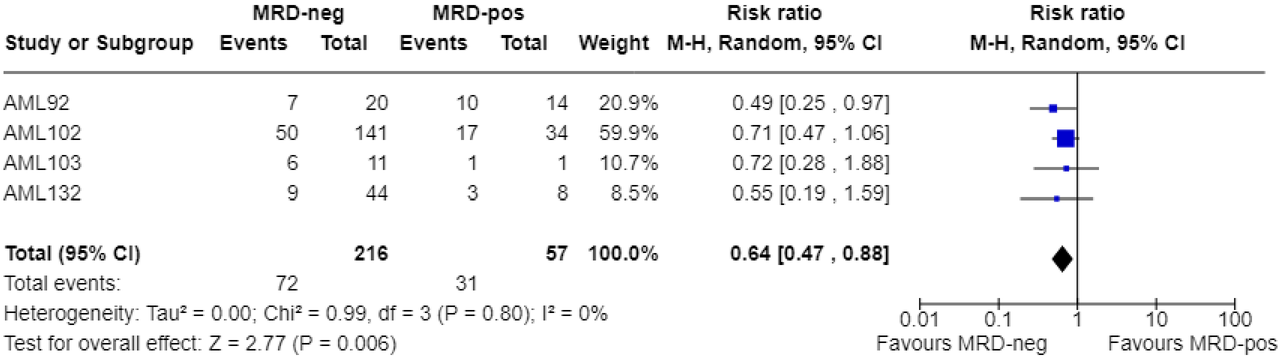

Figure S2: Heterogeneity between studies for 5-year mortality based on MRD status after cycle 2.
